# Redundant and Non-redundant Functions of the AHK Cytokinin Receptors During Gynoecium Development

**DOI:** 10.3389/fpls.2020.568277

**Published:** 2020-10-07

**Authors:** Vincent E. Cerbantez-Bueno, Victor M. Zúñiga-Mayo, J. Irepan Reyes-Olalde, Paulina Lozano-Sotomayor, Humberto Herrera-Ubaldo, Nayelli Marsch-Martinez, Stefan de Folter

**Affiliations:** ^1^Unidad de Genómica Avanzada (UGA-LANGEBIO), Centro de Investigación y de Estudios Avanzados del Instituto Politécnico Nacional (CINVESTAV-IPN), Irapuato, Mexico; ^2^Departamento de Biotecnología y Bioquímica, CINVESTAV-IPN, Irapuato, Mexico

**Keywords:** cytokinin receptors, AHK, gynoecium, transmitting tract, septum, reproduction, fruit

## Abstract

The phytohormone cytokinin is crucial for plant growth and development. The site of action of cytokinin in the plant is dependent on the expression of the cytokinin receptors. In Arabidopsis, there are three cytokinin receptors that present some overlap in expression pattern. Functional studies demonstrated that the receptors play highly redundant roles but also have specialized functions. Here, we focus on gynoecium development, which is the female reproductive part of the plant. Cytokinin signaling has been demonstrated to be important for reproductive development, positively affecting seed yield and fruit production. Most of these developmental processes are regulated by cytokinin during early gynoecium development. While some information is available, there is a gap in knowledge on cytokinin function and especially on the cytokinin receptors during early gynoecium development. Therefore, we studied the expression patterns and the role of the cytokinin receptors during gynoecium development. We found that the three receptors are expressed in the gynoecium and that they have redundant and specialized functions.

## Introduction

Fruit and seed production are largely dependent on sexual plant reproduction. During flower development, androecium and gynoecium development is crucial to allow efficient reproduction. In Arabidopsis, the female reproductive unit is the gynoecium, which develops from the center of the floral primordium as a result of a congenital fusion of two carpels (Bowman et al., 1999; Roeder and Yanofsky, 2006; Ferrándiz et al., 2010). In early developmental stages, the gynoecium grows as a hollow tube and along where the two carpels fuse, a meristematic medial ridge area develops, called carpel margin meristem (CMM) (Reyes-Olalde et al., 2013; Reyes-Olalde and de Folter, 2019). During the mid-stages of gynoecium development, CMM derived tissues such as septum, placenta, ovules, and finally, the transmitting tract develop and start to differentiate. At the mature gynoecium stage, all internal tissues complete their development, and the stigma is ready to receive pollen (Reyes-Olalde et al., 2013). Once the gynoecium is mature and fully developed, the flower opens, ovules become fertilized and fruit development starts. This process combines cell division and differentiation with rapid cell expansion to form a silique that finally will mature and contain the mature seeds inside (Ballester and Ferrándiz, 2017). The correct development of the gynoecium and fruit is coordinated by genetic and hormonal factors (Ferrándiz et al., 2010; Reyes-Olalde et al., 2013; Chávez Montes et al., 2015; Sehra and Franks, 2015; Marsch-Martínez and de Folter, 2016; Deb et al., 2018; Zúñiga-Mayo et al., 2019; Becker, 2020; Cucinotta et al., 2020).

Cytokinin is an adenine-derived hormone that regulates different processes through the life cycle of the plant (Mok and Mok, 2001; Kieber and Schaller, 2018; Márquez-López et al., 2019; Wybouw and De Rybel, 2019). It was first isolated in 1955, as a compound promoting cell division (Miller et al., 1955). In the vegetative phase, cytokinins regulate shoot initiation and growth, cell division, leaf senescence, apical dominance, sink/source relationship, root development, nutrient uptake, phyllotaxis and photomorphogenic development (Werner and Schmülling, 2009; Hwang et al., 2012; Kieber and Schaller, 2014, 2018; Cortleven et al., 2019; Wybouw and De Rybel, 2019). During the reproductive phase, cytokinin has been shown to be involved in organ size and number, and seed yield (Bartrina et al., 2011, 2017; Cucinotta et al., 2016, 2018; Reyes-Olalde et al., 2017; Zúñiga-Mayo et al., 2018). Specifically, during early gynoecium development, the CMM has meristematic activity provided by cytokinin (Bartrina et al., 2011; Marsch-Martínez et al., 2012; Reyes-Olalde et al., 2013; Müller et al., 2017; Reyes-Olalde et al., 2017). Cytokinin activity has also been shown to be present in ovules, septum, transmitting tract, replum, and the valve margins of the gynoecium (Miyawaki et al., 2004; Kinoshita-Tsujimura and Kakimoto, 2011; Bencivenga et al., 2012; Marsch-Martínez et al., 2012; Cheng et al., 2013; Zúñiga-Mayo et al., 2014, 2018). In ovules, the cytokinin function is important for female gametophyte development (Bencivenga et al., 2012; Cheng and Kieber, 2013; Cheng et al., 2013). Furthermore, cytokinin signaling is important for valve margin identity allowing pod shattering (Marsch-Martínez et al., 2012), and for promoting replum development as well (Zúñiga-Mayo et al., 2018). However, a role for cytokinin in other tissues of the gynoecium is still unknown.

Cytokinin receptors are located in the endoplasmic reticulum as well at the plasma membrane where they perceive cytokinin molecules, followed by the activation of a signal transduction pathway, which is a phosphorelay system that reminds us of the bacterial two-component signaling systems (Kakimoto, 1996; Hwang and Sheen, 2001; Schaller et al., 2011; Hwang et al., 2012; Kieber and Schaller, 2018; Romanov et al., 2018; Antoniadi et al., 2020; Kubiasová et al., 2020). Cytokinin is first sensed by a CHASE domain of a histidine kinase receptor (in Arabidopsis, AHK), followed by autophosphorylation of its receiver domain. The signal is then transduced to histidine phosphotransfer proteins (AHP) that shuttle to the nucleus when phosphorylated to activate response regulators (ARR) by phosphorylation (Hwang et al., 2012; El-Showk et al., 2013; Kieber and Schaller, 2014, 2018). In Arabidopsis there are three AHK cytokinin receptors with highly redundant functions, but also with some specialized functions (Heyl et al., 2012; Lomin et al., 2012). The first described cytokinin-responsive AHK gene was WOODEN LEG (WOL), later became known as CYTOKININ RESPONSE1 (CRE1) or ARABIDOPSIS HISTIDINE KINASE4(AHK4; used from here), in a mutant that was insensitive to different exogenous cytokinin applications (Inoue et al., 2001). This was soon followed by the identification of the two other receptors, AHK2 and AHK3, whose primary structures are closely related to AHK4 (Ueguchi et al., 2001). The triple ahk mutant is a miniature plant that almost does not grow and is severely affected in reproduction (Higuchi et al., 2004; Nishimura et al., 2004; Riefler et al., 2006; Kinoshita-Tsujimura and Kakimoto, 2011). Based on genetics, using mutants lacking one, two, or three of the AHK cytokinin receptors, it has been demonstrated that they have redundant functions during plant development (e.g., Higuchi et al., 2004; Nishimura et al., 2004; Franco-Zorrilla et al., 2005; Mähönen et al., 2006; Riefler et al., 2006; López-Bucio et al., 2007; Tran et al., 2007; Gordon et al., 2009; Jeon et al., 2010; Skylar et al., 2010; Kinoshita-Tsujimura and Kakimoto, 2011; Bencivenga et al., 2012; Cheng et al., 2013). However, prominent roles for each one of them in different processes of plant biology have also been shown (reviewed in Heyl et al., 2012; Lomin et al., 2012). AHK2 and AHK3 have been demonstrated to be sufficient to provide the main cytokinin functions during plant development (Kim et al., 2006; Riefler et al., 2006; Hejátko et al., 2009); and AHK4 also showed to play important roles for some developmental processes (Mähönen et al., 2000; Maruyama-Nakashita et al., 2004; Franco-Zorrilla et al., 2005; Pernisova et al., 2018). Important to notice is that the spatio-temporal expression patterns of the receptor encoding genes is different. Although many of the functions of AHK3 are highly overlapping with AHK2, it has been shown that each of them has also specialized functions (Kim et al., 2006; Riefler et al., 2006; Dello Ioio et al., 2007; Bartrina et al., 2017). Furthermore, only in few described cases, either AHK3 or AHK2 showed a stronger redundancy with AHK4 (e.g., Séguéla et al., 2008; Vadassery et al., 2008; Pertry et al., 2009, 2010), however, more overlap must be present because double mutant plants are largely normal versus the ahk triple mutant.

In reproductive tissues, the *ahk2 ahk3 ahk4* triple mutant has shown defects in the formation of the female gametophyte resulting in less fertile ovules. However, this phenotype was only found in the triple mutant, indicating a strong redundant role for the receptors also in reproductive tissues (Kinoshita-Tsujimura and Kakimoto, 2011; Bencivenga et al., 2012; Cheng et al., 2013). In gain-of-function mutants, *AHK2* and *AHK3* were identified as regulators of flowering time and plant longevity. This resulted in increased seed yield due to increased plant longevity and therefore more fruits (Bartrina et al., 2017). In the gynoecium, the three receptors were reported as important for apical-basal patterning. We have shown that using short-term exogenous cytokinin applications, apical-basal patterning defects are induced in wild type gynoecia (for example, reduction in the length of the valves). In *ahk* double mutants, cytokinin insensitivity was observed, depending on the mutant combination used (Zúñiga-Mayo et al., 2014), suggesting that there is no complete redundancy among the cytokinin receptors in the gynoecium. However, to obtain a better understanding of the functions of the cytokinin receptors during gynoecium development, more studies are needed. In this study we addressed these functions. Furthermore, we determined the precise expression patterns of the receptors during gynoecium development. In summary, we observed redundant and specialized functions for the cytokinin receptors in the gynoecium.

## Materials and Methods

### Plant Materials and Growth Conditions

All the reporter lines (*AHK2:GUS, AHK3:GUS*, and *AHK4:GUS*) and mutants (*ahk2-2*, *ahk3-3*, *cre1-12* single, and double mutants) are originally from the Tatsuo Kakimoto laboratory and have been described in Higuchi et al., 2004. All lines are in the *Arabidopsis thaliana* Col-0 background. All lines were germinated in a growth chamber (∼22°C, long day light regime) and then grown under greenhouse conditions during two different season of the year: a “colder” season (16–25°C; ∼11 h of light) and a “hotter” season (18–29°C; ∼13 h of light) in 20°40’36.3” N 101°21.377’ W.

### GUS Analyses

Gynoecia of different developmental stages were dissected and pre-fixed with cold acetone for 20 min, then rinsed and transferred into GUS substrate solution: 50 mM sodium phosphate pH 7, 5 mM K3/K4 FeCN, 0.1% (w/v) Triton X-100, and 2 mM X-Gluc (Gold BioTechnology Inc.). After application of vacuum for 20 min, all samples were incubated at 37°C for 96 h.

### Histology

Tissues were fixed in FAE (3.7% formaldehyde, 5% glacial acetic acid and 50% ethanol) with vacuum (15 min, 4°C) and incubated for 60 min at room temperature. The material was rinsed with 70% ethanol and incubated overnight at 4°C, followed by dehydration in a series of alcohol solutions (70, 85, 95, and 100% ethanol) for 60 min each, and embedded in TechnoVIT, as previously described (Marsch-Martínez et al., 2014). Pictures were taken using a Leica DM6000B microscope (Leica).

### Scanning Electron Microscope Analysis

Fresh tissue samples of gynoecium were visualized in a Zeiss scanning electron microscope EVO40 (Carl Zeiss) using the VPSE G3 or the BSD detector with a 15–20 kV beam.

### Transmitting Tract Analysis

Transmitting tract staining was performed as previously described (Zúñiga-Mayo et al., 2012). In summary, tissue sections were stained with a solution of 0.5% alcian blue (pH 3.1; Sigma-Aldrich) for 25 min and counterstained with a solution of 0.5% neutral red (Sigma-Aldrich) for 5 min. Slides were rinsed in water, air dried, mounted, and observed with a Microscope Axio Imager.Z2 ACR (Zeiss) equipped with a digital camera Axiocam (Zeiss).

### Cytokinin Treatments

Plants were grown, and when they started flowering (approximately 3.5 weeks), inflorescences were treated with the cytokinin 6-Benzylaminopurine (BAP) using the previously described protocol (Zúñiga-Mayo et al., 2014). In summary, one week after bolting, drops were placed on the inflorescences once a day for 10 days with BAP solution (two periods of five days and two days without application between each period), and images of the results were taken 3 to 4 weeks afterward. The BAP solution contains 100 μM 6-Benzylaminopurine (BAP; Duchefa Biochemie) and 0.01% Silwet L-77 (Lehle Seeds). Mock treatments contained only 0.01% Silwet L-77 in distilled water. All plants were grown simultaneously under the same conditions.

### Phenotypic Analyses

Pistil length was determined through the dissection of gynoecia of flowers in stage 13; these gynoecia were measured by image analysis in the ImageJ software (NIH). For ovule number, Arabidopsis gynoecia were dissected from flowers in stage 13 from single and double mutant lines of the cytokinin receptors. These gynoecia were opened along the long axis to obtain a view of the ovules present and the ovule counting was made using ImageJ. Seed number and area analyses were done by collecting seeds from mature and dry fruits. The collected seeds were counted and measured using the ImageJ software. Finally, for fruit length, Arabidopsis fruits at stage 17, obtained from cytokinin receptor single and double mutant were collected and measured in length using the ImageJ software. Statistical analyses were made using one-way ANOVA and Tukey test as a *post hoc* for multiple comparisons (presented in Figure 4). Phenotypes of BAP-treated plants (around 10 plants) were scored as the percentage of gynoecia (*n* = 100–500) presenting ectopic proliferation (presented in Figure 5). All the data were analyzed using R (GNU GLP) or Prism (Graph Pad).

## Results

### Cytokinin Receptors Are Differentially Expressed During Gynoecium Development

**FIGURE 1:**
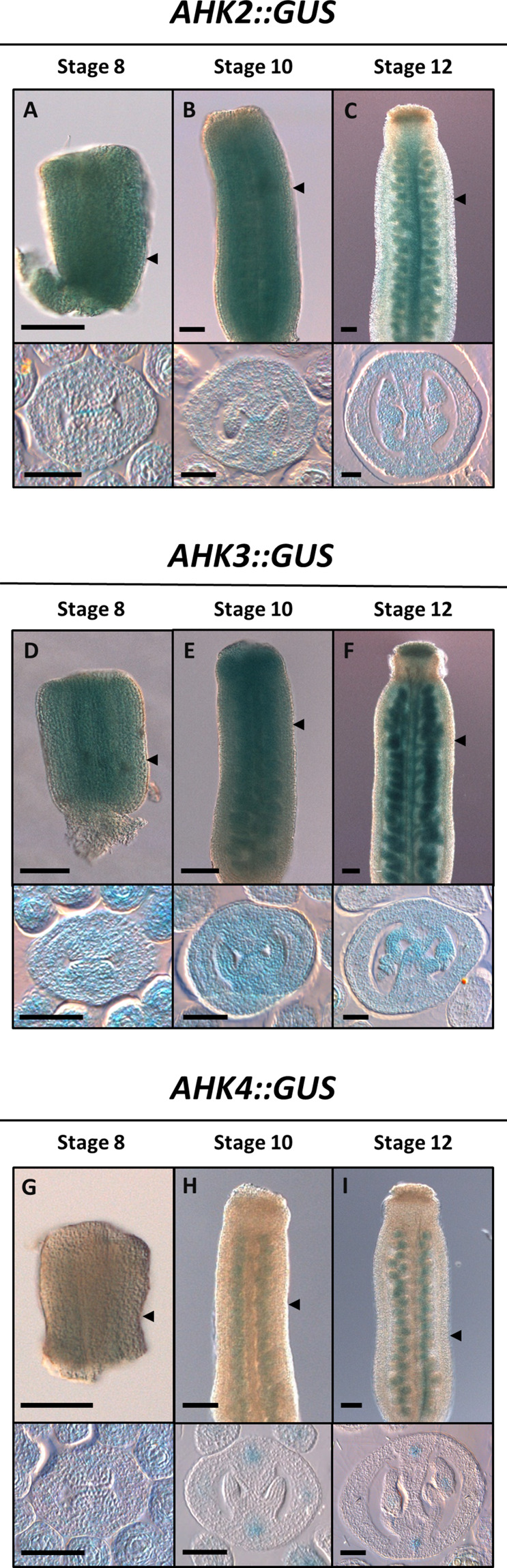
Expression profile of the *AHK* cytokinin receptors during gynoecium development. Longitudinal views (top) and transverse sections (bottom) of the transcriptional fusion reporters *AHK2:GUS* **(A–C),** *AHK3:GUS* **(D–F),** and *AHK4:GUS* **(G–I)** in early **(A, D, G),** mid **(B, E, H),** and mature **(C, F, I)** stages of gynoecium development. Scale bars: 50 μm **(A–I)**. The arrow heads indicate the position of the transverse cut in each longitudinal view.

The expression of the different cytokinin receptors has already been described for vegetative tissues and for some reproductive tissues such as ovules in *Arabidopsis thaliana* (Higuchi et al., 2004; Nishimura et al., 2004; Bencivenga et al., 2012; Cheng et al., 2013). As well, a global overview of the expression patterns of the cytokinin receptors can be obtained from available transcriptomic and proteomic data (Supplementary Figure S1). However, during the reproductive phase, their detailed expression patterns during gynoecium development have not been reported. In this report, we analyzed the expression patterns of the three cytokinin receptors at three developmental time points (early, mid, and almost mature stage) during gynoecium development. In general, based on transcriptional GUS reporter lines, all three receptors are expressed during gynoecium development, however, the expression pattern differs for each gene (Figure 1). We observed *AHK2:GUS* activity ubiquitously in the three gynoecium stages analyzed (Figures 1A–C). In transverse gynoecia sections, a slightly stronger signal at the center of the medial domain is observed, where the two CMMs meet at early stages and later where the transmitting tract is formed during the mature stage. This increased expression in the transmitting tract region in the ovary and style can also be observed in longitudinal gynoecia views, including expression in the stigma (Figure 1C). *AHK3:GUS* activity was observed high and also ubiquitously in the gynoecium in the analyzed stages (Figures 1D–F). Furthermore, in most samples of stages 10–12 gynoecia, an increased GUS signal is observed in placental tissues, funiculi, developing ovules, and the transmitting tract region (Figures 1E,F). Signal is also detected in the style region and clearly in the stigma (Figure 1F). Lastly, for the *AHK4:GUS* reporter, we observed low GUS activity in the complete young gynoecium at stage 8, with some increased signal where the vasculature bundles will be formed (Figure 1G). During the mid and later stages of gynoecium development, *AHK4* expression is clearly observed in foci where the vasculature bundles are located (Figures 1I–H). Furthermore, we also observed expression in developing ovules. No GUS activity was observed in the style or stigma.

In summary, all three cytokinin receptors are expressed during gynoecium development, with *AHK2* and *AHK3* very similar and ubiquitously in all stages, while *AHK4* becomes specifically expressed in the vasculature bundles and in ovules. In addition, a good correlation is observed with the level of expression in the complete carpel (Supplementary Figure S1) compared with our GUS analysis, where *AHK3* has the highest expression, followed by a high expression of *AHK2*, and the lowest expression of *AHK4*.

### AHK Cytokinin Receptors Have Specific Functions During Gynoecium Development

The function of the three cytokinin receptors has been described as strongly redundant, however, for some physiological or developmental processes, some of them have more importance than the others (reviewed by Heyl et al., 2012). As described above, the three cytokinin receptors are expressed during gynoecium development. The expression patterns show overlap, but also show differences, suggesting possible functional redundancy but also specialized functions. To get insight into the function of the AHK cytokinin receptors during gynoecium and fruit development, we analyzed single and double mutants in *Arabidopsis thaliana*. We did not perform any analysis on the triple mutant, which lacks the function of all three cytokinin receptors, and as a result only miniature plants are obtained with a severely reduced reproductive development (data not shown; Higuchi et al., 2004; Nishimura et al., 2004; Riefler et al., 2006; Kinoshita-Tsujimura and Kakimoto, 2011).

**FIGURE 2:**
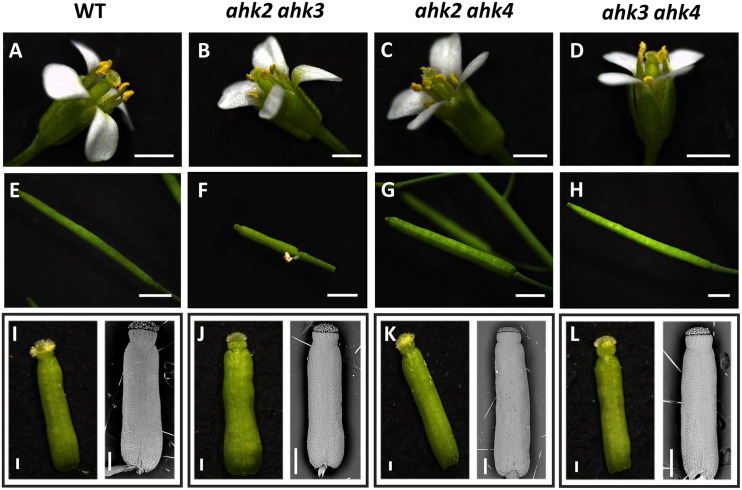
Phenotypes of *ahk* double mutants. Flower phenotypes of wild type **(A)**, *ahk2 ahk3* **(B)**, *ahk2 ahk4* **(C),** and *ahk3 ahk4* **(D)** double mutant plants. Fruit-set phenotypes of wild type **(E),** *ahk2 ahk3* **(F),** *ahk2 ahk4* **(G),** and *ahk3 ahk4* **(H)** double mutant plants. Mature gynoecia phenotypes of wild type **(I),** *ahk2 ahk3* **(J),** *ahk2 ahk4* **(K),** and *ahk3 ahk4* **(L)** double mutant flowers. **I–L** Left side images: stereoscopic images of gynoecia in stage 13. Right side images: scanning electron microscopy images of gynoecia in stage 12. Scale bars: 2 mm **(A–H);** 200 μm **(I–L)**.

Most of the Arabidopsis cytokinin receptor mutants used have been reported as null mutants (Higuchi et al., 2004), except for ahk3-3 where some transcripts have been detected when using an increased number of PCR cycles (Higuchi et al., 2004), although, ahk3-3 is considered a strong loss-of-function mutant. Their phenotypes have been analyzed in different aspects of the plant life cycle (e.g., Higuchi et al., 2004; Nishimura et al., 2004; Riefler et al., 2006; Kinoshita-Tsujimura and Kakimoto, 2011; Bencivenga et al., 2012; Heyl et al., 2012; Cheng et al., 2013), but there is a lack of an integrated analyses of their function during gynoecium and fruit development. We found that in all of our growth conditions, the single mutants *ahk2*, *ahk3*, and *ahk4* developed as normal plants with normal flower and fruit structures as has been reported before (data not shown; Higuchi et al., 2004), and for other mutant alleles as well (Nishimura et al., 2004; Riefler et al., 2006). Likewise, the double mutants *ahk2 ahk4* and *ahk3 ahk4* formed also normal plants with no obvious floral or fruit phenotypes (Figure 2). However, plants of the double mutant *ahk2 ahk3* (*n* = ∼25) were always smaller and shorter, slower in growth, and produced less fruits (data not shown), showing several fruit abortions or very small fruit-like structures (Figure 2F). Thus, we observed that the *ahk2 ahk3* mutant displayed defects in the fruit-set. The reduced general plant size for this double mutant combination has been reported before (Higuchi et al., 2004; Nishimura et al., 2004; Riefler et al., 2006).

**FIGURE 3:**
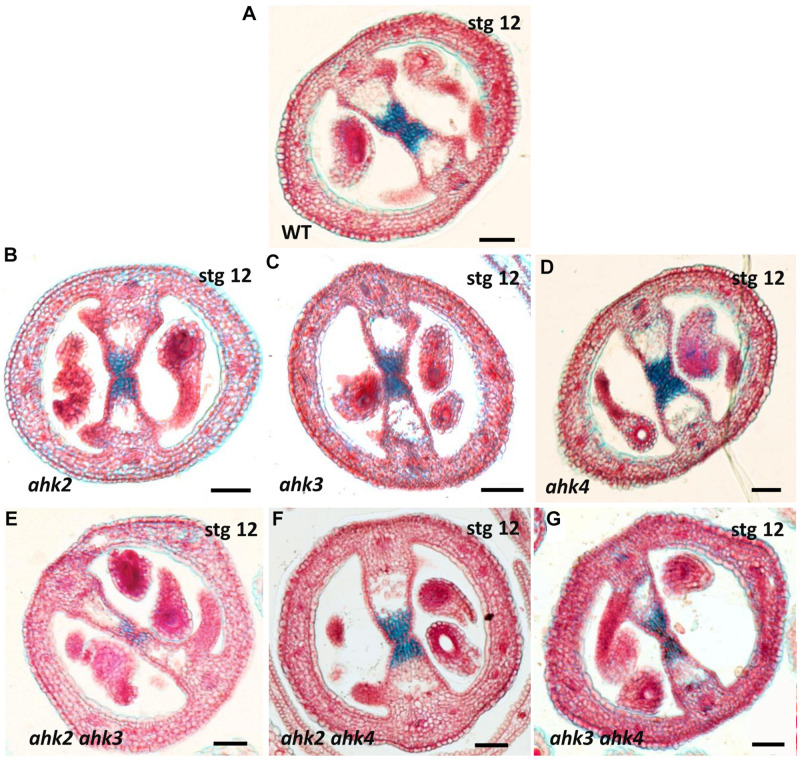
Stained transverse sections of mature gynoecia of single and double *ahk* mutants. **(A)** Wild type gynoecium with a normal transmitting tract (blue stained cells). **(B–D)** Transverse sections of *ahk* single mutants. **(B)** *ahk2* single mutant with a normal transmitting tract. **(C)** *ahk3* single mutant with a normal transmitting tract. **(D)** *ahk4* single mutant with a normal transmitting tract. **(E–G)** Transverse sections of *ahk* double mutants. **(E)** *ahk2 ahk3* double mutant with defects in the transmitting tract. **(F)** *ahk2 ahk4* double mutant with a normal transmitting tract. **(G)** *ahk3 ahk4* double mutant with mild defects in the transmitting tract. Scale bars: 50 μm **(A–G)**.

**FIGURE 4:**
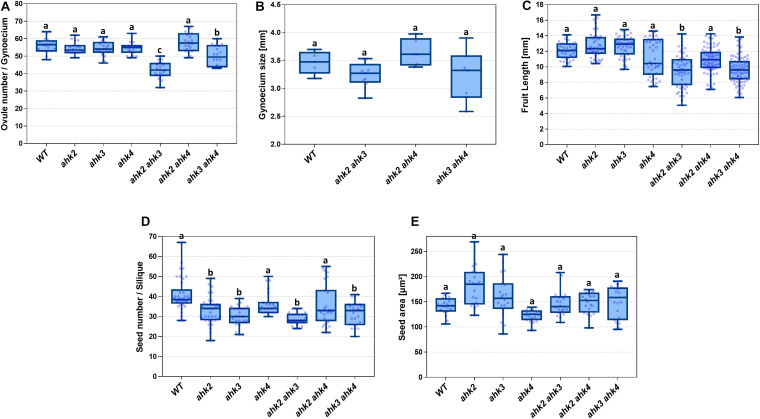
Phenotypic analyses of single and double *ahk* mutants compared to wild type. **(A)** Ovule number of single and double mutants. **(B)** Gynoecium size of double mutants. **(C)** Fruit length of single and double mutants. **(D)** Seed number of single and double mutants. **(E)** Seed area of single and double mutants. Sample numbers: **(A)** *n* = 20 in all lines; **(B)** WT, *n* = 5; *ahk2 ahk3*, *n* = 9; *ahk2 ahk4*, *n* = 6; *ahk3 ahk4*, *n* = 7; **(C)** WT, *n* = 41; *ahk2*, *n* = 41; *ahk3*, *n* = 43; *ahk4*, *n* = 43; *ahk2 ahk3*, *n* = 56; *ahk2 ahk4*, *n* = 82; *ahk3 ahk4*, *n* = 77; **(D)** WT, *n* = 38; *ahk2*, *n* = 37; *ahk3*, *n* = 27; *ahk4*, *n* = 23; *ahk2 ahk3*, *n* = 27; *ahk2 ahk4*, *n* = 27; *ahk3 ahk4*, *n* = 26; **(E)** *n* = 20 for all lines. **(A–E)** One-way ANOVA and Tukey as *post hoc* were applied; differences in groups were determined by *p* value comparison to wild type (WT) and are represented with different letters. **(A) a** = no significant difference; **b** = p ≤ 0.05; **c** = *p* ≤ 0.0001; **(B–E) a** = no significant difference; **b** = *p* ≤ 0.0001.

As mentioned above, all double mutant combinations form flowers with normal appearing gynoecia (Figures 2A–D, I–L), indicating that a single AHK receptor is enough for the overall floral development and that the cytokinin receptors are redundant in this process. Interestingly, as mentioned above, plants of the *ahk2 ahk3* double mutation were affected in fruit-set, which could be due to the growth conditions or developmental defects occurring during gynoecium development. No affected pollen development has been reported for the *ahk* double mutants, only reduced pollen grain production and reduced fertility in the ahk triple mutant (Nishimura et al., 2004; Kinoshita-Tsujimura and Kakimoto, 2011). To obtain insight in pollen grain germination and pollen tube growth, we observed pollen tube movement through the ovary using Aniline Blue staining. The results showed that pollen grains can germinate, and pollen tube growth seems normal in all our mutant combinations (Supplementary Figure S2). Therefore, we focused on the gynoecium and performed more detailed morphological and histological analysis of mutant gynoecia. First, we made thin transverse sections and observed the internal structures of the mature gynoecium by staining the sections with alcian blue and neutral red. In sections of wild type mature gynoecia, all internal tissues are correctly formed, including the transmitting tract, which can be seen by the blue stained cells in the middle of the septum (Figure 3). The same correct patterning was observed in the single *ahk* mutants compared to the wild type phenotype (Figures 3A–D), indicating again a redundant function for the cytokinin receptors. However, while transverse sections of gynoecia of the *ahk2 ahk4* double mutant presented also a normal patterning (Figure 3F), some alterations in patterning in the *ahk2 ahk3* and *ahk3 ahk4* double mutants were observed (Figures 3E,G). Gynoecia of these double mutants, especially of *ahk2 ahk3*, showed an apparent reduced transmitting tract, indicated by a reduction of cells with blue staining in the middle of the septum. These alterations could affect fertility, leading to reduced seed-set. Alterations in seed-set often lead to reduced fruit size and/or production, which we observed in the *ahk2 ahk3* double mutant. Reduced seed-set can also occur when ovule development is affected. It has been reported that ovule development (i.e., female gametophytic development) is not impaired in single or double cytokinin receptor mutant combinations, but in the ahk triple mutant both ovule development and ovule number are clearly affected (Kinoshita-Tsujimura and Kakimoto, 2011; Bencivenga et al., 2012; Cheng et al., 2013), with some differences in penetrance based on the alleles used (Cheng and Kieber, 2013). Therefore, we did not analyze ovule development, though, we did count the number of ovules present in gynoecia. Compared to the wild type and single *ahk* mutants, we found a statistically significant reduction in ovule number in the double mutants *ahk2 ahk3* and *ahk3 ahk4* (Figure 4A). The *ahk2 ahk3* mutant developed 32–50 ovules per gynoecium (*n* = 20), the *ahk3 ahk4* mutant 32–60 ovules per gynoecium (*n* = 20), while wild type developed 48–64 ovules per gynoecium (*n* = 20). Based on the mean, the double mutants have a reduction in ovule number of around 25% and 11%, respectively. Reduced ovule number is often reflected in reduced gynoecium size (Cucinotta et al., 2020). Indeed, the *ahk2 ahk3* and *ahk3 ahk4* mutants developed slightly reduced gynoecia with a size ranging from 2.8–3.5 and 2.5–3.9 mm, respectively. However, they were not statistically significant different from the size of wild type gynoecia (3.1–3.6 mm) (Figure 4B).

In summary, all double mutants produced mature gynoecia. However, detailed analyses showed alterations in internal tissues. Transmitting tract development seem to present defects, and ovule number was also negatively affected. The results support that the three cytokinin receptors have partially redundant roles during gynoecium development, with AHK3 having the most prominent role based on the phenotypes observed in the different double mutant combinations.

### Mutants in AHK Receptors Present Fruit and Seed Alterations

Generally, defects in tissue formation during gynoecium development have an effect on the fruit, after pollination. So, we were expecting aberrant phenotypes when analyzing the fruits of the *ahk2 ahk3* and *ahk3 ahk4* mutants. As mentioned before, the growth of *ahk2 ahk3* mutant plants is affected, and in our growth conditions these plants developed less fruits. However, the fruits that were formed, were used for further analyses. Fruit development relies on the combination of cell division and differentiation, with rapid cell expansion to form a mature silique with fully developed seeds inside (Ferrandiz, 2002). So, fruit length and the correct formation of seeds are features that indicate that this developmental process occurred correctly. We found alterations in fruit length for some of the mutants analyzed (Figure 4C). The *ahk2 ahk3* and *ahk3 ahk4* double mutants developed shorter fruits with ranges of 5–14.2 and 6–13.8 mm in length, respectively. The statistical analyses showed that they are significantly different from the wild type fruit length (10–14.1 mm, *n* = 42). Based on the mean, the double mutants have a reduction in fruit length of around 20%. The *ahk4* and *ahk2 ahk4* mutants also presented some shorter fruits, but they were not statistically significant different from the wild type. Thus, the observed alterations in the gynoecium in the *ahk2 ahk3* and *ahk3 ahk4* double mutants seem to be reflected in altered fruit development.

Next, the number of seeds in mature fruits of the different mutants was determined. As expected, the double *ahk2 ahk3* and *ahk3 ahk4* mutant fruits contained less seeds (Figure 4D). The wild type fruits developed 28–67 seeds per fruit, while the double mutants *ahk2 ahk3* and *ahk3 ahk4* developed 24–34 and 20–41 seeds per fruit, respectively. The statistical analyses showed that these differences in seed number per fruit are significant. Based on the mean, the double mutants have a reduction in seed number of around 30% and 20%, respectively. Surprisingly, the *ahk2* and *ahk3* single mutants also had a statistically significant reduced seed number (18–49, *n* = 37 and 21–39, *n* = 27; respectively), compared to the wild type (Figure 4D). Based on the mean, a reduction of 19% and 26%, respectively. It has been reported that reduced cytokinin sensitivity can lead to bigger seeds (Riefler et al., 2006). Therefore, we measured also seed size. Indeed, we found some seeds in *ahk2*, *ahk3*, *ahk2 ahk3*, and *ahk3 ahk4* mutants increased in size compared to wild type seeds (Figure 4E). However, these results were not statistically significant different.

As mentioned above, the alterations in the gynoecium seem to be reflected as a phenotype in fruit development and seed number for *ahk2 ahk3* and *ahk3 ahk4*. Surprisingly, we also observed differences in seed number for single *ahk* mutants that seemed to have no alterations in gynoecium development.

### ahk Mutants Respond Differently to Exogenous Cytokinin Application

Another strategy to address the contribution of the different cytokinin receptors during gynoecium development is using a pharmacological assay. We have shown before that the repeated application of exogenous cytokinin alters gynoecium development. Depending on the duration of the cytokinin applications, tissue overproliferation causing ectopic outgrowths from the replum of the gynoecium upon long-term application (Marsch-Martínez et al., 2012; Reyes-Olalde et al., 2017) or gynoecia with apical-basal patterning defects upon short-term application are observed (Zúñiga-Mayo et al., 2014).

**FIGURE 5:**
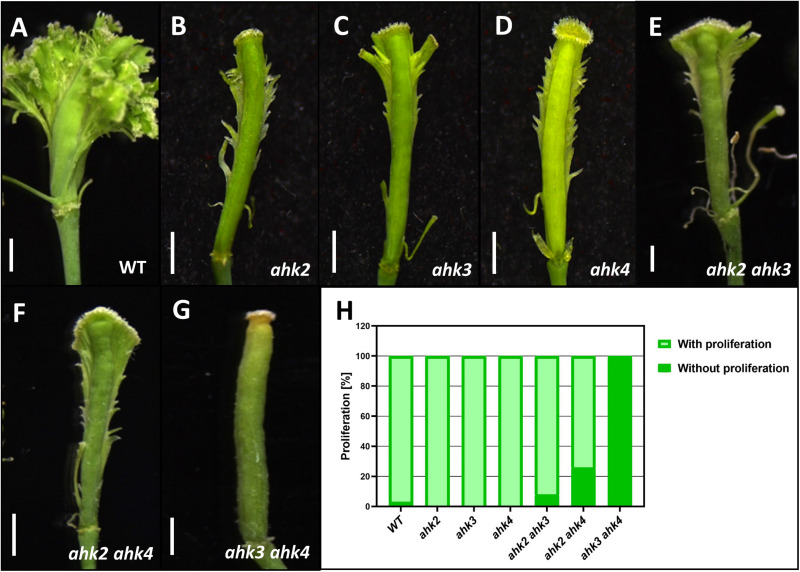
Phenotypes of gynoecia three to four weeks after receiving a cytokinin-treatment for ten days. **(A)** Wild type gynoecium with ectopic tissue proliferation. **(B–D)** Gynoecia of single mutants *ahk2* **(B)**, *ahk3* **(C)**, and *ahk4* **(D)** with reduced ectopic tissue proliferation. **(E, F)** Gynoecia of double mutants *ahk2 ahk3* **(E)**, *ahk2 ahk4* **(F)** with reduced ectopic tissue proliferation. **(G)** Gynoecium of the double mutant *ahk3 ahk4* without ectopic tissue proliferation. **(H)** Percentage of gynoecia with ectopic proliferation of each genotype. WT, *n* = 438; *ahk2*, *n* = 100; *ahk3*, *n* = 100; *ahk4*, *n* = 100; *ahk2 ahk3*, *n* = 224; *ahk2 ahk4*, *n* = 288; *ahk3 ahk4*, *n* = 500. Scale bars: 1 mm **(A–G)**.

Here, we applied the long-term cytokinin treatment to the single and double *ahk* mutants and compared them to wild type plants. In wild type gynoecia, as seen before, abundant ectopic outgrowths were observed (Figure 5). However, in the single *ahk* mutants, a strong reduction of ectopic outgrowths was observed, meaning that the three cytokinin receptors participate in producing the full response to the exogenous cytokinin application (Figures 5B–D). For the double mutants, *ahk2 ahk3* and *ahk2 ahk4* showed both also a strong reduction of ectopic outgrowths, however, in the *ahk3 ahk4* double mutant a lack of response to cytokinin was observed (Figure 5G). In line with this, the *ahk3 ahk4* double mutant also showed no apical-basal gynoecium defects upon a short-term cytokinin application (Zúñiga-Mayo et al., 2014).

In summary, the cytokinin receptors play a redundant role in the response to exogenous cytokinin application. Removing one receptor already shows a strong reduction in the response and based on the effects of the treatment in double mutant combinations, we conclude that AHK2 alone is not sufficient to produce any response.

## Discussion

Cytokinin signaling is important for many aspects during plant development. During gynoecium and fruit development, cytokinin signaling also fulfills essential roles (e.g., Bartrina et al., 2011; Marsch-Martínez et al., 2012; Zúñiga-Mayo et al., 2014, 2018; Marsch-Martínez and de Folter, 2016; Durán-Medina et al., 2017; Müller et al., 2017; Reyes-Olalde et al., 2017; Cucinotta et al., 2020; Di Marzo et al., 2020). In this study we addressed the expression and function of the three AHK cytokinin receptors during gynoecium development in Arabidopsis, complementing other studies on reproductive development such as anther and ovule development (Kinoshita-Tsujimura and Kakimoto, 2011; Bencivenga et al., 2012; Cheng and Kieber, 2013; Cheng et al., 2013). In general, our results indicate redundant but also specific functions for the AHK receptors during gynoecium and fruit development (Table 1), which are further discussed in continuation.

**TABLE 1:** AHK redundant and specific functions during gynoecium and fruit development.

**Function**	**Cytokinin receptor**		
	**AHK2**	**AHK3**	**AHK4**
CMM positive meristematic activity	**	***	*
Transmitting tract development	**	***	*
Ovule development	**	***	*
Affecting fruit length	**	***	*
Affecting seed production	**	***	*
Gynoecium vasculature development	*	*	*
Affecting auxin transport	*	**	**

Key: functional importance indicated with asterisks (*), more asterisks means more important.

### The AHK Receptors Have Redundant but Also Specific Functions

In general, plant growth is normal for most mutants except for the *ahk2 ahk3* double mutant, where plant growth was quite affected and plant size was reduced by around half. The reduction in plant size due to the absence of these two receptors has been reported, indicating an important and prominent role in growth and maintenance of meristematic activity in the shoot apical meristem (Higuchi et al., 2004; Nishimura et al., 2004; Riefler et al., 2006). When the third receptor, AHK4, is also not functional, root development is also affected, and severe dwarfed plants are the result. The triple mutant produces almost no flowers and the few that are produced are sterile (Higuchi et al., 2004; Nishimura et al., 2004; Riefler et al., 2006; Kinoshita-Tsujimura and Kakimoto, 2011). In our study we did not use the ahk2 ahk3 ahk4 triple mutant due to the lack of enough flowers to analyze. In our study, the *ahk2 ahk3* mutant displayed reduced plant size, but was also affected in fruit and seed production. These phenotypes were not reported before (Nishimura et al., 2004; Riefler et al., 2006). A possible explanation is that we used the ahk2-2 and ahk3-3 alleles, and in other reports different alleles were used. Differences in phenotypic severity in ahk alleles has been discussed (Cheng and Kieber, 2013). The alleles that we used have been reported as presenting stronger effects in plant development (Higuchi et al., 2004; Cheng et al., 2013). We used the alleles reported in Higuchi et al. (2004), though to our knowledge the authors did not describe the effect we observed. However, the images in their report suggests that the same phenotype was present (Supplementary Figure S11 in Higuchi et al., 2004). The phenotypes we observed during gynoecium development, especially in the *ahk2 ahk3* double mutant, that are further discussed below, indicate that AHK2 and AHK3 play roles in plant reproduction and fruit production. Therefore, our analyses complement the knowledge of the roles of the CRE family members.

### AHK2 and AHK3 Positively Regulate Meristematic Activity During Gynoecium Development, With AHK3 Having a Prominent Role

As mentioned, no obvious morphological differences were observed in the general external aspect of the gynoecium in the *ahk* double mutants, but we noticed developmental alterations in tissues in the medial domain inside the gynoecium. In the different analyses, we found that not all double mutant combinations developed a normal gynoecium and fruit, supporting that the functions of CRE family members are not completely redundant. The *ahk2 ahk3* and *ahk3 ahk4* double mutants showed developmental defects in gynoecia and fruits. Although this could suggest that the three receptors are involved, the phenotypes in the *ahk2 ahk3* mutant were more severe, evidenced as reduced transmitting tract development, and a reduction in ovule number. This is concordant with the overall aspect of the semi-dwarfed plants, where we found that *ahk2 ahk3* was the only mutant that was affected in fruit production. This supports that the function of AHK2 and AHK3 play a predominant role in gynoecium and fruit development. Moreover, since the *ahk2 ahk4* double mutant has no remarkable phenotypes, it is reasonable to think that AHK3 has a prominent role in these developmental processes, followed by AHK2. The presence of a functional AHK3 alone allows the development of an entire gynoecium with normal size and internal tissues. This coincides with other findings, where AHK3 is redundant in function with AHK2, but plays a prominent role in some other processes (Kim et al., 2006; Riefler et al., 2006). This prominent and overlapping role is consistent with the expression patterns of *AHK2* and *AHK3* in the gynoecium. Based on transcriptional fusion lines, we observed that the expression of *AHK2* and *AHK3* is ubiquitous in the gynoecium, but more intense in tissues in the medial domain of the gynoecium. The expression pattern of *AHK4* is very different, mostly localized in foci marking the vasculature bundles, though also weakly in ovules. In more detail, while *AHK2* maintained intense expression in the very central area of the medial domain during development, *AHK3* was always more intense than *AHK2* and was even more intense in placenta, funiculi, ovules and transmitting tract tissues when the gynoecium reached a mature stage.

The medial domain of the gynoecium at early developmental stages is characterized by being mitotically active. At early stages, this medial domain is denominated the carpel margin meristem (CMM), a tissue with meristematic activity that gives rise to all the internal tissues of the gynoecium (Reyes-Olalde et al., 2013; Reyes-Olalde and de Folter, 2019). The CMM and medial tissues that develop from it, present a high cytokinin signaling response output based on the TCS:GFP cytokinin response reporter (Marsch-Martínez et al., 2012; Reyes-Olalde et al., 2013, 2017; Müller et al., 2017). Based on the overlap of TCS signal with the expression patterns of AHK2 and AHK3, it is likely to assume that AHK2 and AHK3 are the two main cytokinin receptors mediating the positive meristematic activity and patterning function orchestrated by cytokinin in the medial domain of the gynoecium. In line with this, all tissues derived from the CMM are clearly affected in the *ahk2 ahk3* double mutant. In support of these findings, we have observed similar phenotypes in CMM-derived tissues in the type-B *arr1 arr10 arr12* triple mutant (Reyes-Olalde et al., 2017), which is severely affected in cytokinin signaling (Mason et al., 2005; Argyros et al., 2008; Ishida et al., 2008). Furthermore, a meristematic activity-promoting function of AHK2 and AHK3 has been reported before in shoot apical meristem maintenance (Higuchi et al., 2004; Nishimura et al., 2004; Riefler *et al.*, 2006). Finally, it is known that ovule number, which is also dependent on CMM activity, is positively regulated by cytokinin signaling (Bartrina et al., 2011; Cucinotta et al., 2016, 2018; Reyes-Olalde et al., 2017; Zúñiga-Mayo et al., 2018). Accordingly, we observed a mild but significant reduction in ovule number in *ahk2 ahk3* and to a lesser extent in the *ahk3 ahk4* double mutant.

In summary, the data suggest that AHK3 and AHK2 have the prominent promoting role in meristematic activity in the medial domain of the gynoecium.

### AHK2 and AHK3 Affect Seed Production

The *ahk2 ahk3* and *ahk3 ahk4* mutants showed a reduction in ovule number, which in turn leads to reduced seed production. Indeed, both double mutants produced less seeds. The *ahk2 ahk4* double mutant showed no significant difference in seed production. The impaired development of tissues in the medial domain during gynoecium development in the *ahk2 ahk3* and *ahk3 ahk4* double mutants, had an effect on the reproductive competence of the plant, evidenced in reduced seed yield. Interestingly, we already noticed a reduction in seed number in the single *ahk2* and *ahk3* mutants under our growth conditions. We observed no difference in ovule number in these single mutants, and previous reports have suggested that ovule development is not affected in them, only in the triple mutant (Kinoshita-Tsujimura and Kakimoto, 2011; Bencivenga et al., 2012; Cheng et al., 2013). Furthermore, based on analysis in the ahk triple mutant, it has been reported that the AHK receptors play also a role in pollen-pistil interaction (Kinoshita-Tsujimura and Kakimoto, 2011). Despite the redundancy among the receptors, AHK2 and AHK3 are involved in female gametophyte development, ovule number, and internal tissue development of the gynoecium, so probably the observed reduction in seed number is a combined effect. Interestingly, recently it has been described that *AHK2* and *AHK3* are regulators of seed yield using gain-of-function mutants, although this function was attributed to the fact that the plants presented increased longevity and therefore generated more siliques (Bartrina et al., 2017). According to this and our results, we can confirm that cytokinin, mainly via the AHK2 and AHK3 receptors, has an effect on seed production.

### AHK4 Function in Gynoecium and Fruit Development Is Redundant but Necessary

With our study on gynoecium development, we demonstrated the importance of the cytokinin receptors AHK2 and AHK3. The role for AHK4 was less obvious. Compared with *AHK2* and *AHK3*, the *AHK4* receptor has a very different expression pattern, as reported also for other tissues (Higuchi et al., 2004; Nishimura et al., 2004). It marked where the vasculature will develop and maintained this pattern as foci in the place where the vasculature bundles are located in the gynoecium. It has been demonstrated that cytokinin is important for vasculature development. The AHK receptors are redundant in this function, with AHK4 playing an important role (Mähönen et al., 2000, 2006). The presence of the *AHK4* cytokinin receptor in this area, overlapping with *AHK2* and *AHK3* expression, suggests that it has a redundant function for vasculature development also in the gynoecium. In summary, the AHK4 function in gynoecium development and patterning seems to be less evident from the double mutant studies, though based on the severe phenotypes in the triple mutant, AHK4 also supports cytokinin function in the gynoecium and reproductive development.

### AHK3 and AHK4 Are Needed to Respond to Exogenous Cytokinin

As shown before, long-term cytokinin applications lead to tissue overproliferation causing ectopic outgrowths from the replum of the gynoecium (Marsch-Martínez et al., 2012; Reyes-Olalde et al., 2017). To test the role of the receptors in this phenomenon, we used the same methodology with a high cytokinin concentration to assure a clear effect would be observed, not really the effect of cytokinin *per se*. Despite the very high concentration, the observed effect is not random and clearly depends on the presence of specific actors in the cytokinin signaling pathway. First, all the single cytokinin receptor mutants showed a decreased response to exogenous cytokinin, meaning that each receptor is part of providing the full cytokinin-response as observed in wild type gynoecia. Second, the *ahk2 ahk3* and *ahk2 ahk4* double mutant also presented reduced cytokinin-response, comparable to the single mutants. Remarkably, the *ahk3 ahk4* double mutant showed a lack of response, since no ectopic tissue proliferation was observed. Previously, we have observed a similar lack of cytokinin-response in the form of absence of apical-basal patterning defects after a short-time cytokinin application (Zúñiga-Mayo et al., 2014). This is also consistent with reports on callus induction assays, where after the triple mutant, the *ahk3 ahk4* double mutant is most affected (Higuchi et al., 2004). Also, in shoot regeneration assays, specially AHK4 seems to be important to sense exogenous cytokinins (Inoue et al., 2001; Nishimura et al., 2004; Pernisova et al., 2018). Interestingly, cytokinin-regulated expression of the meristem stem cell niche markers *WUSCHEL* (*WUS*) and *WOX5* is dependent on AHK4 (Gordon et al., 2009; Pernisova et al., 2018). These are genes that have not been reported to be expressed in the gynoecium, however, perhaps other *WOX* family members in the gynoecium are dependent on AHK4. In summary, our results demonstrate that AHK3 and AHK4 are important for the response to exogenous cytokinin in the gynoecium. The AHK2 receptor alone is not sufficient to provide a response to an exogenous cytokinin-application. These results are likely to stay the same at lower concentrations of cytokinin. The CRE family members have been described as having different affinities to different types of cytokinins (Spíchal et al., 2004; Romanov et al., 2006; Stolz et al., 2011; Lomin et al., 2015; Pernisova et al., 2018). Based on ligand-binding studies, AHK2 and AHK4 have been demonstrated to have a similar affinity range to the cytokinin benzylaminopurine (BA or BAP; used in this study), but AHK3 has up to 10x less affinity to this cytokinin (Stolz et al., 2011; Lomin et al., 2015), suggesting that the difference in response is not due to a lack of cytokinin perception. Another important aspect is the expression patterns of the receptors. To obtain a cytokinin-response, the receptor should be expressed in the correct tissue. However, the expression patterns of *AHK2* and *AHK3* are not identical but are very similar, making this not the most obvious explanation. Note, we analyzed transcriptional fusion reporters and we cannot conclude that this will reflect the exact protein localization. On the other hand, as we have reported before, the ectopic tissue proliferation in response to BAP is linked to the regulation of auxin transport via the type-B ARR transcription factors and PIN3 (Reyes-Olalde et al., 2017), it might suggest that this phenomenon is mostly dependent on the AHK3 and AHK4 receptors. These two receptors have also been reported to be important in the root meristem for cytokinin-regulated auxin transport via PIN auxin transporters (reviewed in Hwang et al., 2012). In addition, the type-B ARR transcription factors ARR1 and ARR12, functioning at the end of the cytokinin signaling pathway, are involved in the phenomenon, because in single mutants a strong reduction and in the *arr1 arr12* double mutant a lack of response to exogenous cytokinin in the gynoecium is observed (Reyes-Olalde et al., 2017).

In summary, with this work we can conclude that the AHK cytokinin receptors have redundant and specialized functions in the gynoecium of Arabidopsis. Future work on AHK receptors during gynoecium development in other plant species would be interesting.

## Data Availability Statement

The raw data supporting the conclusions of this article will be made available by the authors, without undue reservation.

## Author Contributions

SF and NM-M conceived and designed the research. VC-B, VZ-M, JR-O, PL-S, and HH-U performed the research. VC-B and SF wrote the manuscript. SF supervised the research. All authors approved the manuscript.

## Conflict of Interest

The authors declare that the research was conducted in the absence of any commercial or financial relationships that could be construed as a potential conflict of interest.

## Supplementary Material

Supplementary Figure 1 RNA and protein levels of the AHK receptors in Arabidopsis thaliana. **(A)** Expression levels (RNA) among different plant tissues. **(B)** Expression levels (RNA) in the flower. **(C)** Expression levels (protein) among different flower tissues. **(D)** Expression levels (protein and RNA) in the carpel. Data obtained from the ARS database (Zhang et al., 2020; http://ipf.sustech.edu.cn/pub/athrna/) **(A,B)**; and from the ATHENA database (Mergner et al., 2020; http://athena.proteomics.wzw.tum.de:5002/master_arabidopsisshiny/) **(C,D)**.

Supplementary Figure 2 Longitudinal view of pollen tube growth in mature gynoecia of single and double *ahk* mutants after pollination. **(A–G)** Gynoecia in stage 13 with pollen tube growth, visualized with Aniline Blue staining. **(A)** WT gynoecium with pollen tube growth. **(B–D)** Gynoecia of single mutants *ahk2* **(B)**; *ahk3* **(C)**, and *ahk4* **(D)**, with pollen tube growth. **(E–G)** Gynoecia of double mutants *ahk2 ahk3* **(E)**; *ahk2 ahk4* **(F)**, and *ahk3 ahk4* **(G)**, with pollen tube growth. Seemingly normal pollen tube growth is observed in all genotypes. Scale bars = 200 μm **(A–G)**.
