# Supplementary material for: Redundant and Non-redundant Functions of the AHK Cytokinin Receptors During Gynoecium Development
Source: Front Plant Sci. 2020 Oct 7;11:568277. doi: 10.3389/fpls.2020.568277 (PMC7575793; doi:10.3389/fpls.2020.568277)
Supplement: Supplementary Figure 1 — RNA and protein levels of the AHK receptors in Arabidopsis thaliana. (A) Expression levels (RNA) among different plant tissues. (B) Expression levels (RNA) in the flower. (C) Expression levels (protein) among different flower tissues. (D) Expression levels (protein and RNA) in the carpel. Data obtained from the ARS database (Zhang et al., 2020; http://ipf.sustech.edu.cn/pub/athrna/) (A,B); and from the ATHENA database (Mergner et al., 2020; http://athena.proteomics.wzw.tum.de:5002/master_arabidopsisshiny/) (C,D). [file Data_Sheet_1.PDF]

Supplementary Material

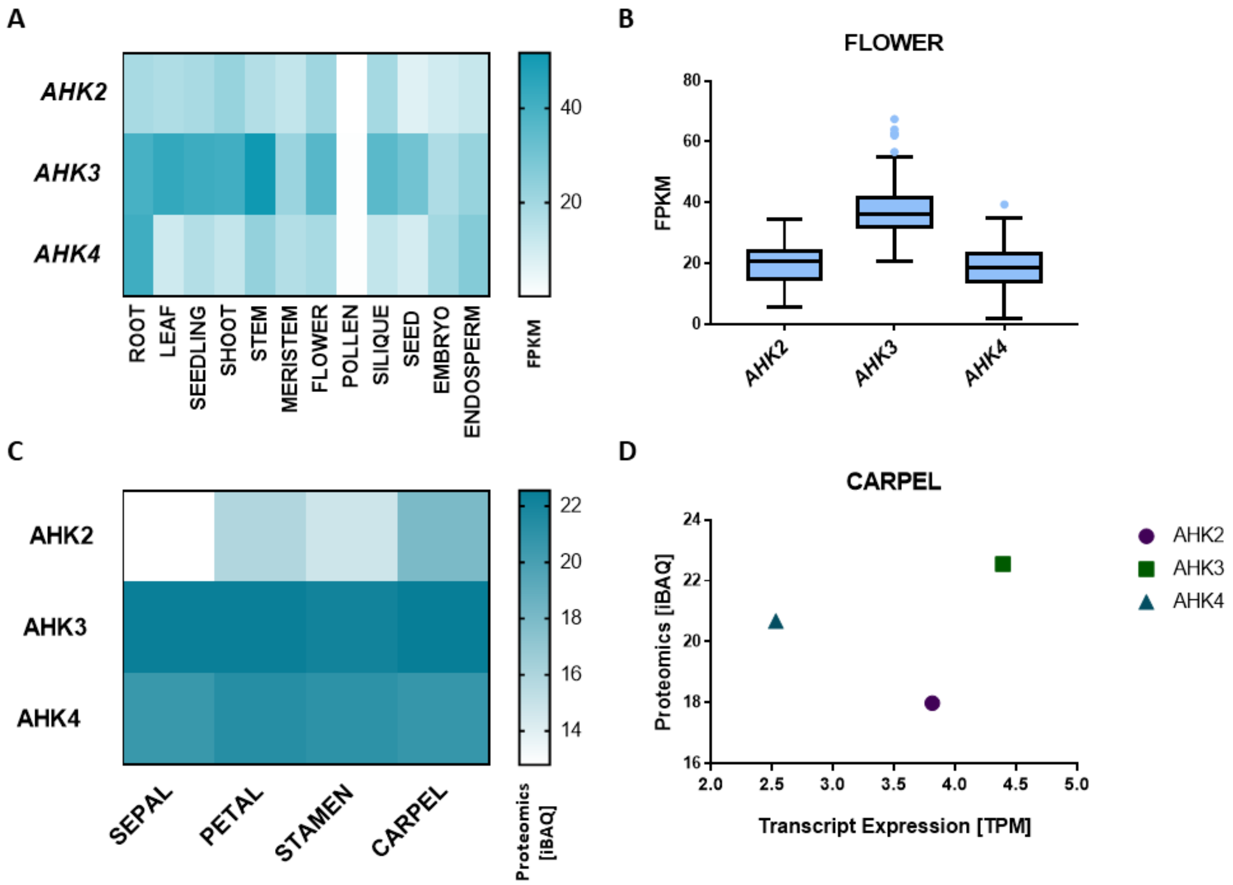

Supplementary Figure 1. RNA and protein levels of the AHK receptors in *Arabidopsis thaliana*. **(A)** Expression levels (RNA) among different plant tissues. **(B)** Expression levels (RNA) in the flower. **(C)** Expression levels (protein) among different flower tissues. **(D)** Expression levels (protein and RNA) in the carpel. Data obtained from the ARS database (Zhang et al., 2020; http://ipf.sustech.edu.cn/pub/athrna/) **(A, B)**; and from the ATHENA database (Mergner et al., 2020; http://athena.proteomics.wzw.tum.de:5002/master_arabidopsisshiny/) **(C, D)**.
