# Supplementary material for: Redundant and Non-redundant Functions of the AHK Cytokinin Receptors During Gynoecium Development
Source: Front Plant Sci. 2020 Oct 7;11:568277. doi: 10.3389/fpls.2020.568277 (PMC7575793; doi:10.3389/fpls.2020.568277)
Supplement: Supplementary Figure 2 — Longitudinal view of pollen tube growth in mature gynoecia of single and double ahk mutants after pollination. (A–G) Gynoecia in stage 13 with pollen tube growth, visualized with Aniline Blue staining. (A) WT gynoecium with pollen tube growth. (B–D) Gynoecia of single mutants ahk2 (B); ahk3 (C), and ahk4 (D), with pollen tube growth. (E–G) Gynoecia of double mutants ahk2 ahk3 (E); ahk2 ahk4 (F), and ahk3 ahk4 (G), with pollen tube growth. Seemingly normal pollen tube growth is observed in all genotypes. Scale bars = 200 μm (A–G). [file Data_Sheet_2.PDF]

Supplementary Material

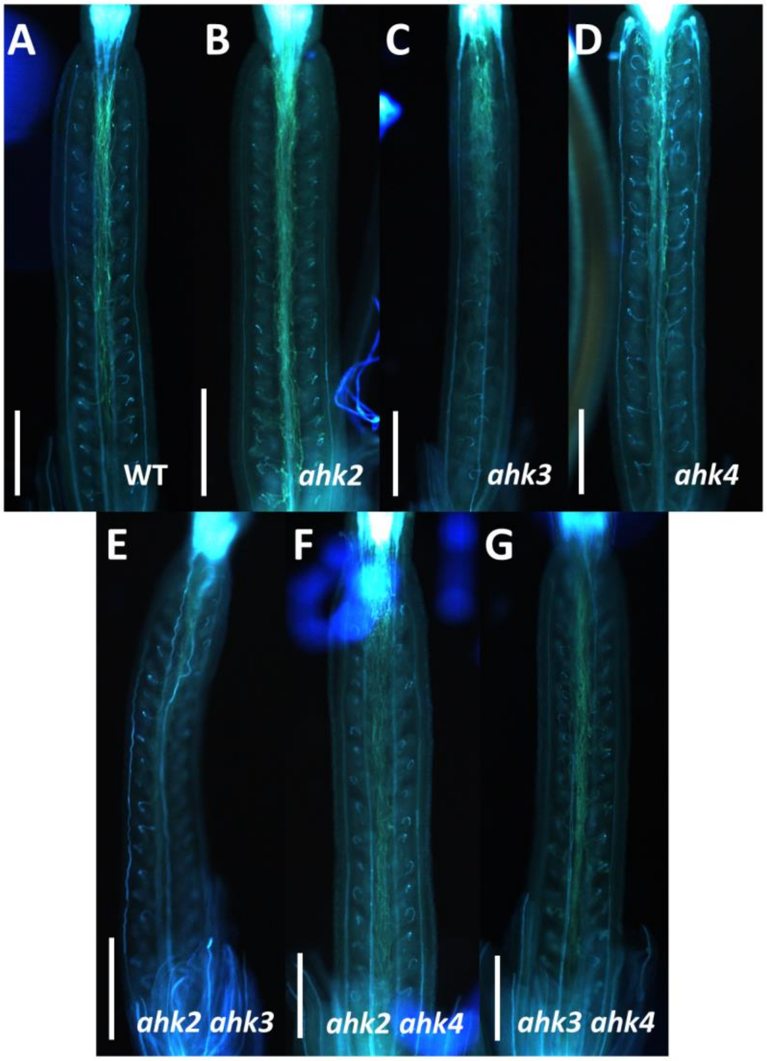

Supplementary Figure 2. Longitudinal view of pollen tube growth in mature gynoecia of single and double *ahk* mutants after pollination. **(A-G)** Gynoecia in stage 13 with pollen tube growth, visualized with Aniline Blue staining. **(A)** WT gynoecium with pollen tube growth. **(B-D)** Gynoecia of single mutants *ahk2* **(B)**; *ahk3* **(C)** and *ahk4* **(D)**, with pollen tube growth. **(E-G)** Gynoecia of double mutants *ahk2 ahk3* **(E)**; *ahk2 ahk4* **(F)** and *ahk3 ahk4* **(G)**, with pollen tube growth. Seemingly normal pollen tube growth is observed in all genotypes. Scale bars = 200 µm **(A-G)**.
